# Prevalence of Erectile Dysfunction in Patients with Diabetes Mellitus and Its Association with Body Mass Index and Glycated Hemoglobin in Africa: A Systematic Review and Meta-Analysis

**DOI:** 10.1155/2020/5148370

**Published:** 2020-01-18

**Authors:** Wondimeneh Shibabaw Shiferaw, Tadesse Yirga Akalu, Yared Asmare Aynalem

**Affiliations:** ^1^Lecturer of Nursing, Department of Nursing, College of Health Science, Debre Berhan University, Debre Berhan, Ethiopia; ^2^Lecturer of Nursing, Department of Nursing, College of Health Science, Debre Markos University, Debre Markos, Ethiopia

## Abstract

**Background:**

Mortality and morbidity in patients with diabetes mellitus (DM) are attributed to both microvascular and macrovascular complications. However, there is a significant amount of variation in the primary studies on DM regarding the prevalence of erectile dysfunction (ED) in Africa. Therefore, this study was aimed to estimate the pooled prevalence of ED patients with DM and its association with body mass index (BMI) and glycated hemoglobin in Africa.

**Methods:**

PubMed, Web of Science, Cochrane Library, Scopus, PsycINFO, African Journals Online, and Google Scholar were searched for studies that looked at ED in DM patients. A funnel plot and Egger's regression test were used to determine publication bias. The *I*^2^ statistic was used to check heterogeneity between the studies. DerSimonian and Laird random-effects model was applied to estimate the pooled effect size. The subgroup and meta-regression analyses were conducted by country, sample size, and year of publication. Sensitivity analysis was deployed to see the effect of a single study on the overall estimation. STATA version 14 statistical software was used for the meta-analysis.

**Result:**

A total of 13 studies with 3,501 study participants were included in this study. We estimated that the pooled prevalence of ED in patients with DM in Africa was 71.45% (95% CI: 60.22–82.69). Diabetic patients whose BMI was ≥30 kg/m^2^ were 1.26 times more likely to develop ED (AOR = 1.26; 95% CI: 0.73–2.16) and whose glycated hemoglobin was <7% were 7% less likely to develop ED (AOR = 0.93; 95% CI: 0.5–5.9), although they were not significantly associated with ED.

**Conclusions:**

The prevalence of ED in DM patients in Africa remains high. Therefore, situation-based interventions and country context-specific preventive strategies should be developed to reduce the prevalence of ED among patients with DM.

## 1. Introduction

Diabetes is a major public health problem, increasingly affecting millions of people across the globe [1]. Approximately 425 million people suffered from diabetes mellitus (DM) globally in 2017; by 2045, this is projected to rise to 629 million [2]. The prevalence of DM in Africa also continues to rise, thus imposing an extraburden on Africa's healthcare systems [3]. Mortality and morbidity in patients with DM are often the result of both micro- and macrovascular complications. One common and yet underestimated complication of DM is erectile dysfunction (ED) [4]. Indeed, it is estimated that the global prevalence of ED should reach 322 million by 2025 [5]. ED is the inability to achieve and maintain an erection sufficient to permit satisfactory sexual intercourse [6]. It may result from psychological, neurologic, hormonal, or arterial impairment or from a combination of these factors [7].

The pathophysiology of ED in DM is related to multiple mechanisms, including endothelial dysfunction, the accumulation of advanced glycation end products, oxidative stress, and autonomic neuropathy [4, 8]. For instance, diabetes may affect the cavernous nerve terminals and endothelial cells, resulting in a deficiency in neurotransmitters [9].

Large differences have been reported on the prevalence of ED in patients with DM in different studies. For instance, ED has been reported in 49% of the male population in England [10], 35.8% in Italy [11], 65.4% in Korean [12], 86.1 in Saudi Arabia [13], 31% in Kuwait [14], 38.9% in India [15], 77.1% in South Africa [16], and 67.9% in Ghana [17]. The risk factors for ED are multifactorial and complex. Studies suggest that risk factors for ED in patients with DM include hypertension [14, 16, 18, 19], heart disease [18], cigarette smoking [14, 16, 18–20], low education level [14], increasing body mass index (BMI) [14, 15, 17], poor glycemic control [14, 17, 21], age above or equal to 50 years [14, 15, 17, 20, 22], metabolic syndrome [23], testosterone deficiency [24], increasing duration of diabetes [22], presence of depressive symptoms [15], high income [17], fat-rich diets [25], and other diabetic complications [26].

Patients with ED often suffer from poor quality of life [19, 27], anxiety when sexual ability declines [28], mutual mistrust, general unhappiness, and fear of losing support from their partner [29]. However, effectively controlling the disease can reduce some of these negative effects. For instance, several studies have shown that about one-third of men with ED show improvement in sexual function based on lifestyle interventions, such as diet, exercise and weight loss, cessation of smoking, counseling, and appropriate glycemic control through diet [30, 31]. Different primary studies in Africa show the magnitude of ED as a health issue in the region. However, variation was observed among these studies. Therefore, this systematic review and meta-analysis aimed to estimate the pooled prevalence of ED in patients with DM and its association with BMI and glycated hemoglobin in Africa.

## 2. Methods

### 2.1. Data Sources and Literature Search Strategy

Electronic databases, such as PubMed, Google Scholar, African Journals Online, Scopus, Web of Science, PsycINFO, and the Cochrane library, were independently and systematically searched by the authors. In addition, a manual search of gray literature and other related articles were deployed to identify additional relevant research. Data from the International Diabetic Federation (IDF) were also searched and used. This search involved articles published from January 1, 1990, to September 4, 2019. The searches were restricted to full texts, free articles, human studies, and English-language publications. Authors were contacted for full texts of their articles through e-mail, if necessary. The search was conducted using the following terms and phrases: “erectile dysfunction,” “sexual dysfunction,” “impotence,” “diabetes mellitus,” and “Africa.” Boolean operators like “AND” and “OR” were used to combine search terms. Particularly, to fit the advanced PubMed database, the following search strategy was used (“erectile dysfunction” OR “impotence” OR “sexual dysfunction”) AND (“diabetes mellitus”) AND (“Africa”).

### 2.2. PECOS Guide

#### 2.2.1. Type of Participants

This review considered studies that included adult male patients aged 18 years or older who had been diagnosed with DM.

#### 2.2.2. Type of Exposure

In this meta-analysis, patients with DM whose BMI was ≥30 kg/m^2^ and glycated hemoglobin was >7% were considered as exposed variable to estimate its effect on ED.

#### 2.2.3. Comparison

In this meta-analysis, patients with DM whose BMI was 18.5–24.9 kg/m^2^ and glycated hemoglobin was <7% were considered as control variable for ED.

#### 2.2.4. Study Outcome

The outcome of this study was the prevalence of ED among men with DM.

### 2.3. Types of Study Designs Used in This Review

This systematic review included observational studies such as retrospective or prospective cohort studies, and cross-sectional and case-control studies, where ED among DM patients has been reported.

### 2.4. Eligibility Criteria

Studies were included in the meta-analysis if they adhered to the following guidelines: (1) all observational studies needed to report the prevalence of ED; (2) articles must be published in peer-reviewed journals or gray literature; (3) articles must be published in English between 1990 and 2019; and (4) studies must examine an African population and include male participants. Studies were excluded if (1) they were not fully accessible; (2) studies with duplicated citation; (3) they possessed a poor quality score as per the stated criteria; (4) they failed to determine the desired outcome (i.e., ED); or (5) they included only females.

### 2.5. Selection and Quality Assessment

Two independent investigators screened the title and abstract of all of the potential studies to be included in our analysis. Data were extracted from each of these studies using the standardized data extraction format prepared in a Microsoft Excel worksheet by the three authors independently. For each article, we extracted data regarding the names of the authors, year of publication, study area, study design, sample size, data collection year, sampling technique, diagnostic criteria used for ED, reported prevalence with its 95% confidence interval (CI), and information regarding the associated factors. When some information was missing, first or corresponding authors of the article were contacted at least twice in a month to obtain the variables of interest. The quality of each included study was assessed using the Newcastle–Ottawa scale (NOS) [32]. This scale has several key criteria including the representativeness of the sample, response rate, measurement tool used, comparability of the subject, and the appropriateness of the statistical test used to analyze the data. Studies were included in the analysis if they scored ≥5 out of 10 points in three domains of ten modified NOS components for a cross-sectional study [33]. Any disagreements at the time of data abstraction were resolved by discussion and consensus (Supplementary file 1).

### 2.6. Statistical Analysis

To obtain the pooled effect size, a meta-analysis using the random-effects DerSimonian and Laird model was performed [34, 35]. Heterogeneity across the included studies was checked using the chi-square-based Q test and the *I*^2^ statistical test with a value of ≥75% for the first test and *p* < 0.05 indicating the presence of significant heterogeneity [35]. To investigate the sources of heterogeneity, meta-regression and subgroup analyses were performed. Potential publication bias was assessed by visual inspection of a funnel plot. In addition, an Egger regression test was conducted, and *p* ≤ 0.05 was considered statistically significant for the presence of publication bias [36]. Sensitivity analysis was deployed to see the effect of a single study on the overall effect estimation. The meta-analysis was performed using the STATA version 14 statistical software for Windows [37].

### 2.7. Data Synthesis and Reporting

To estimate the overall prevalence of ED in patients with DM, the Preferred Reporting Items for Systematic Reviews and Meta-Analyses (PRISMA) guideline was used [38]. The entire process of study screening, selection, and inclusion were described with the aid of a flow diagram. Results were presented using forest plots and summary tables.

## 3. Results

### 3.1. Search Results

**Figure 1:**
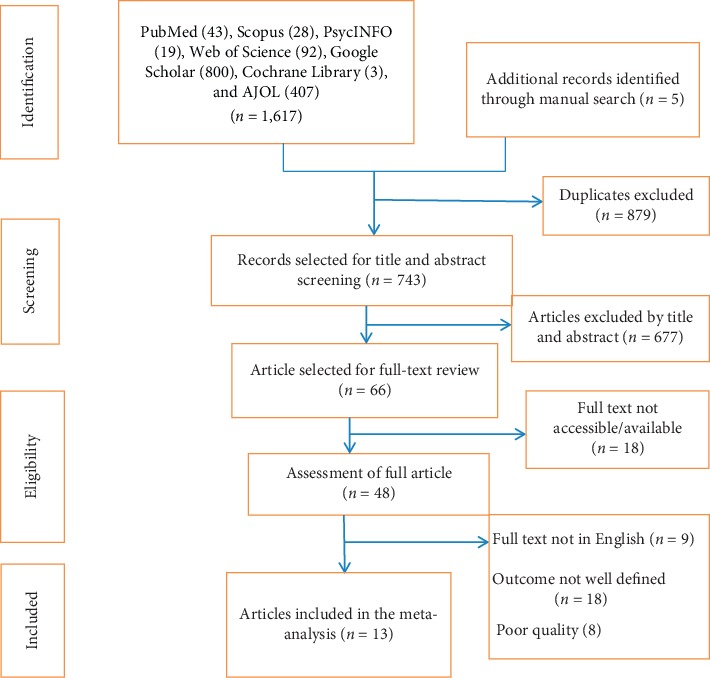
PRISMA flow chart for study selection.

In the first step of our search, 1,622 studies were retrieved. About 1,617 studies were found from seven international databases, and the remaining 5 were through a manual search. The databases included PubMed (43), Scopus (28), Google Scholar (800), Web of Science (317), Cochrane Library (3), PsycINFO (19), and African Journals Online (407). Of these, 879 duplicate records were identified and removed. From the remaining 743 articles, 677 articles were excluded after reading the titles and abstracts based on the predefined eligibility criteria. Finally, 66 full-text articles were assessed for their eligibility. Based on the predefined criteria and quality assessment, only 13 articles were included for the final analysis (Figure 1).

### 3.2. Baseline Characteristics of the Included Studies

In the current meta-analysis, a total of 13 studies with 3,501 study participants were included to estimate the pooled prevalence of ED among DM patients. With respect to study design, the majority (95%) of the studies included were cross sectional. The studies varied substantially in sample size, ranging from 70 to 599. The highest prevalence (95%) of ED was reported in a study conducted in South Africa [39], whereas the lowest prevalence (36%) was reported in a study also conducted in South Africa [40]. Overall information regarding the prevalence of ED in DM patients was obtained from various countries in Africa. Two of the studies involved participants from Nigeria [41, 42], two from Ethiopia [43, 44], two from South Africa [39, 40], one each from Guinea [45], Egypt [21], Kenya [46], Ivory coast [47], Zimbabwe [48], Tanzania [49], and Ghana [17].

Regarding the sampling technique employed, 7 of the studies [17, 21, 41, 42, 46–48] used the consecutive sampling technique to select study participants. However, two studies [45, 49] did not report their sampling methods. With respect to the tools used to assess ED in DM patients, 10 studies [21, 41–49] used the International Index of Erectile Function, two [39, 40] used the Sexual Health Inventory for Men, and one study [17] used the Golombok Rust Inventory of Sexual Satisfaction. The quality score of each primary study, based on the Newcastle–Ottawa quality score assessment, was moderate to high for all 13 articles assessed (Table 1).

### 3.3. Prevalence of Erectile Dysfunction

**Figure 2:**
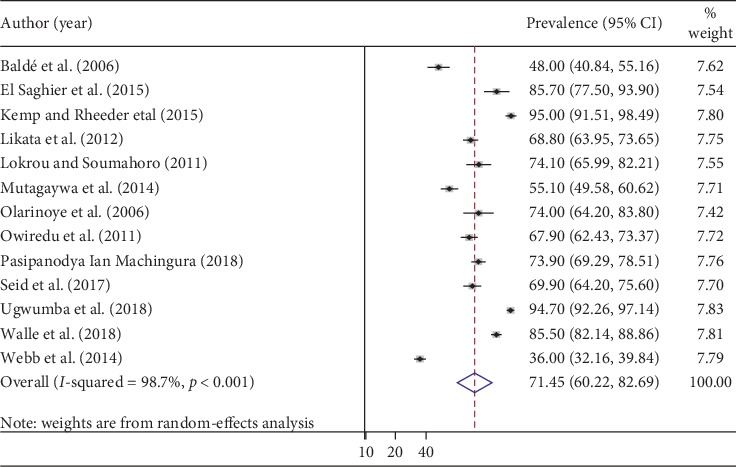
Forest plot of the prevalence of erectile dysfunction in patients with diabetes mellitus.

Our meta-analysis using the random-effects model showed that the estimated overall prevalence of ED in patients with DM was 71.45% (95% CI: 60.22–82.69) with a significant level of heterogeneity (*I*^2^ = 98.7%; *p* < 0.001) (Figure 2).

### 3.4. Subgroup Analysis

To overcome the presence of heterogeneity, subgroup analysis using country, publication year, sample size, and sampling technique was done. Based on this, the prevalence of ED was found to be 84.92% in Nigeria, 73.33% in studies published since 2010, and 74.51% in studies with a sample size less than 300 (Table 2).

### 3.5. Meta-Regression Analysis

To identify the sources of heterogeneity, meta-regression analysis was undertaken by considering year of publication, sample size, and sampling technique. However, our results showed that the covariates were not significantly associated with the presence of heterogeneity (Table 3).

### 3.6. Sensitivity Analysis

**Figure 3:**
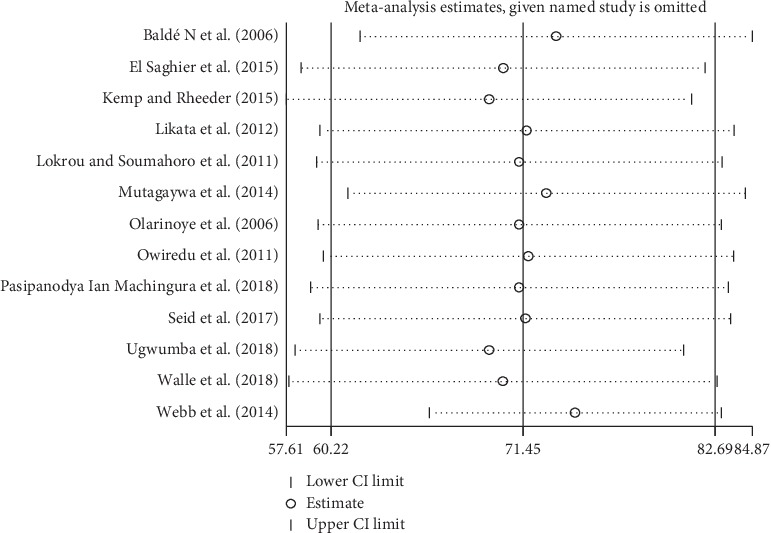
Sensitivity analysis of the 13 studies.

To evaluate the effect of an individual study on the pooled effect size, sensitivity analysis was conducted. Sensitivity analyses using the random-effects model revealed that no single study influenced the overall prevalence of ED in DM patients (Figure 3).

### 3.7. Publication Bias

**Figure 4:**
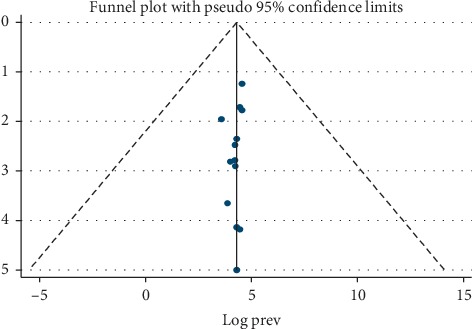
Funnel plot for the presence of publication bias among the 13 included studies.

We found that there was no publication bias among the included studies, as depicted by the symmetrical distribution of our funnel plot (Figure 4) and the results from our Egger's regression test (*p*=0.226).

### 3.8. BMI

**Figure 5:**
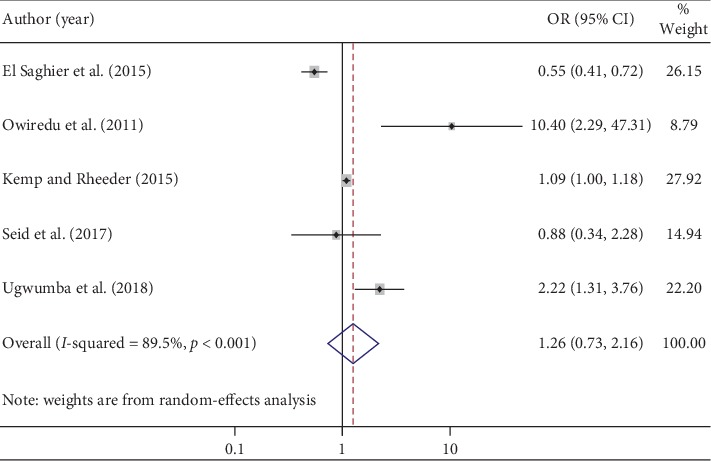
The pooled effects of body mass index on erectile dysfunction.

The pooled effects of five studies showed that BMI of ≥30 kg/m^2^ was not statistically associated with ED in patients with DM (Figure 5). The heterogeneity test (*I*^2^ = 89.5%) showed significant evidence of variation across studies. The evidence from Egger's regression test showed that there was no publication bias (*p*=0.807).

### 3.9. Glycosylated Hemoglobin (Hga1c)

**Figure 6:**
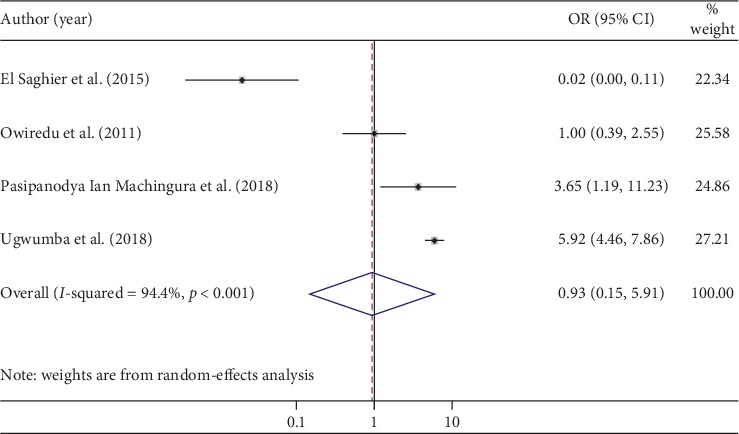
The pooled effects of glycated hemoglobin ≥7% on erectile dysfunction.

According to our current meta-analysis, those who had normal glycated hemoglobin were 7% less likely to develop ED compared with those who had a glycated hemoglobin value ≥7%, although this association was not statistically significant (OR: 0.93; 95% CI: 0.15–5.91) (Figure 6). The heterogeneity test (*I*^2^ = 94.4%) showed significant evidence of variation across studies. The result of Egger's regression test showed no evidence of publication bias (*p*=0.147).

## 4. Discussion

The prevalence of ED in this meta-analysis was estimated to be 71.45% in patients with DM. This indicates that ED is highly prevalent in diabetic patients and is an inadequately controlled complication of DM in African populations. Hence, a serious and multifactorial approach is required to manage diabetes-related ED, with emphasis being placed on treatment adherence, self-care, and health information dissemination.

Our estimated prevalence of ED in DM patients in Africa is substantially higher than that reported in a systematic review and meta-analysis conducted at the global level, which sets the prevalence at 52.5% [50]. This variation could be justified by the different diagnostic criteria for ED, differences in methodology, and population characteristics.

The subgroup analysis in this study showed that the pooled prevalence of ED among diabetic patients in Nigeria was 84.92% (95% CI: 64.66–105.17), which was the highest among the African nations examined, including the prevalence in Ethiopia (77.88%; 95% CI: 62.59–93.16) and South Africa (65.5%; 95% CI: 7.9–123.3). This variation might be due to differences in health-seeking behavior between the populations, differences in the diagnostic methods, and sociodemographic characteristics.

Though we identified two factors (BMI and glycated hemoglobin) that may be related to ED in DM patients, a BMI of ≥30 kg/m^2^ and glycated hemoglobin of ≥7% were not significantly associated with ED among diabetic patients. Specifically, we found that those who had BMI of ≥30 kg/m^2^ were 26% more likely to develop ED, although the increase was not statistically significant. This is supported by studies conducted on populations in Vietnam [51] and Brazil [52]. Similarly, we found that those who had glycosylated hemoglobin of <7% were 7% less likely to develop ED, although the decrease was not statistically significant. This too was supported by studies done on Korean [12] and Chinese [53] populations.

This study has clinical implications in that the high prevalence of ED in DM patients should guide health care professionals to increase patient awareness of DM complications and design preventive measures and treatments to improve patient quality of life. In addition, identifying associated factors may help health care professionals treat DM patients with ED during their clinical care.

The meta-analysis conducted in this study has several limitations that should be considered in future research. First, it is difficult to determine if the results from various countries is representative of the entire content because no data were found for all of Africa. Second, only English articles were considered, and thus, we could be missing important data published in other languages. Moreover, as mentioned previously, we did not identify all of the potential predictors of ED among patients with DM. Therefore, further study is needed to identify associated factors for the development of ED among patients with DM may be necessary, such as age, duration of diabetes, preexisting illness, sedentary lifestyle, and smoking.

## 5. Conclusion and Recommendations

This study revealed that the prevalence of ED remains high in DM patients in Africa. That said, the prevalence of ED differed by country. Therefore, situation-based interventions and country context-specific preventive strategies should be developed to reduce the prevalence of ED among patients with DM.

## Figures and Tables

**Table 1:** Baseline characteristics of the included studies.

First author	Publication year	Country	Study design	Sample size	Prevalence of ED (95% CI)	Data collection year	Data collection tool	Sampling technique	Quality score (10)
Baldé et al. [45]	2006	Guinea	Cross-sectional	187	48 (40.8, 55.2)	NR	International Index of Erectile Function	NR	7
El Saghier et al. [21]	2015	Egypt	Cross-sectional	70	85.7 (77.5, 93.9)	March 2014 to January 2015	International Index of Erectile Function-5	Consecutive	7
Kemp and Rheeder [39]	2015	South Africa	Cross-sectional	150	95 (91.5, 98.5)	NR	The Sexual Health Inventory for Men	Simple random	7
Likata et al. [46]	2012	Kenya	Cross-sectional	350	68.8 (63.9, 73.6)	NR	International Index of Erectile Function	Consecutive	7
Lokrou and Soumahoro [47]	2011	Ivory coast	Cross-sectional	112	74.1 (65.9, 82.2)	NR	International Index of Erectile Function	Consecutive	7
Mutagaywa et al. [49]	2014	Tanzania	Cross-sectional	312	55.1 (49.5, 60.6)	May to December 2011	International Index of Erectile Function	NR	7
Olarinoye et al. [41]	2006	Nigeria	Cross-sectional	77	74 (64.2, 83.8)	NR	International Index of Erectile Function	Consecutive	7
Owiredu et al. [17]	2011	Ghana	Cross-sectional	300	67.9 (62.4, 73.3)	November 2010 to March 2011	Golombok Rust Inventory of Sexual SATISFACTION	Consecutive	7
Pasipanodya Ian Machingura [48]	2018	Zimbabwe	Cross-sectional	348	73.9 (69.3, 78.5)	October 23, 2013 to July 9, 2014	International Index of Erectile Function	Consecutive	6
Seid et al. [43]	2017	Ethiopia	Cross-sectional	249	69.9 (64.2, 75.6)	January 1–February 30, 2016	International Index of Erectile Function	Systematic random	8
Ugwumba et al. [42]	2018	Nigeria	Cross-sectional	325	94.7 (92.2, 97.1)	September 2016 to December 2017	International Index of Erectile Function	Consecutive	7
Walle et al. [44]	2018	Ethiopia	Cross-sectional	422	85.5 (82.1, 88.8)	January to March 2016	International Index of Erectile Function	Systematic	6
Webb et al. [40]	2014	South Africa	Cluster randomized control trial	599	36 (32.1, 39.8)	NR	Sexual Health Inventory For Men	Systematic	8

NR; not reported.

**Table 2:** The prevalence of erectile dysfunction and estimates of heterogeneity when publications are divided into different subgroups.

Sub-group	Category	No. of studies included	Sample size	Prevalence (95% CI)	*p* value	*I* ^2^
By year of publication	Before 2010	2	264	60.78 (35.3, 86.25)	<0.001	94.3
	2010 and above	11	3,237	73.33 (61.23, 85.44)	<0.001	98.8

Sample size	<300	6	845	74.51 (59.54, 89.5)	<0.001	97
	≥300	7	2,656	68.88 (52.17, 85.58)	<0.001	99.2

Country	Nigeria	2	402	84.92 (64.66, 105.17)	<0.001	93.8
	South Africa	2	749	65.51 (7.9, 123.32)	<0.001	99.8
	Ethiopia	2	671	77.88 (62.59, 93.16)	<0.001	95.3
	Others	7	1,679	67.52 (59.45, 75.59)	<0.001	92.2

Sampling technique	Systematic	3	996	83.63 (71.6, 95.66)	<0.001	97.1
	Simple random	1	70	85.7 (77.5, 93.9)	0.1	0.1
	Consecutive	7	2,136	67.22 (50.2, 84.24)	<0.001	98.5
	Not specified	2	299	60.98 (35.40, 86.55)	<0.001	95.5

**Table 3:** Meta-regression analysis for the included studies to identify the sources of heterogeneity.

Variables	Category	Coef.	Std. err.	*t*	*p* < |*t*|	(95% conf. interval)
By year of publication	Before 2010(reference)	0.421	1.106	0.38	0.771	(−2.043 to 2.88)
	2010 and above					

Sample size	<300 (reference)	−0.041	0.487	−0.08	0.934	(−1.126 to 1.044)
	≥300					

Sampling technique	Systematic	0.614	1.267	0.51	0.628	(−2.35 to 3.63)
	Simple random	0.254	1.85	0.14	0.895	(−4.13 to 4.64)
	Consecutive	0.202	1.219	0.17	0.873	(−2.68 to 3.08)
	Not specified (reference)					

## Abbreviations

AOR: Adjusted odds ratio
CI: Confidence interval
DM: Diabetes mellitus
ED: Erectile dysfunction
IDF: International Diabetic Federation
PRISMA: Preferred Reporting Items for Systematic Reviews and Meta-Analyses.

## Supplementary Materials

Supplementary Materials Supplementary file 1: methodological quality assessment of cross-sectional studies using modified Newcastle–Ottawa Scale (NOS).
