# Supplementary material for: Prevalence of Erectile Dysfunction in Patients with Diabetes Mellitus and Its Association with Body Mass Index and Glycated Hemoglobin in Africa: A Systematic Review and Meta-Analysis
Source: Int J Endocrinol. 2020 Jan 18;2020:5148370. doi: 10.1155/2020/5148370 (PMC7201640; doi:10.1155/2020/5148370)
Supplement: Supplementary Materials — Supplementary file 1: methodological quality assessment of cross-sectional studies using modified Newcastle–Ottawa Scale (NOS). [file 5148370.f1.pdf]

Methodological quality assessment

Supplementary file 1: Methodological quality assessment of cross-sectional studies using modified Newcastle - Ottawa Scale (NOS)

**First author**	Criteria								
	**Selection**				**Comparability**		**Outcome**		
	**Representativeness of the sample**	**Sample size**	**Non – responders**	**Ascertainment of exposure/risk factor**	**The study controls for the most important factor**	**The study control for any additional factor**	**Assessment of the outcome**	**Statistical test**	**Total score (10)**
Balde N etal [44]	A*	**B***	**B***	**A***	**-**	**B***	A*	**A***	**7**
El Saghier EO et al [22]	B*	**B***	**A***	**A***	**A***	**-**	A*	**A***	**7**
Kemp T etal [38]	**A***	**A***	**A***	**A***	**A***	**-**	A*	**A***	**7**
Likata GMU etal [45]	A*	**B***	**B***	**A***	**-**	**B***	A*	**A***	**7**
Lokrou A etal [46]	B*	**B***	**A***	**A***	**A***	**-**	A*	**A***	**7**
Mutagaywa RK etal [48]	**B***	**A***	**A***	**B***	**A***	**A***	A*	**A***	**7**
Olarinoye J etal [40]	**A***	**A***	**A***	**A***	**A***	**B***	A*	**A***	**7**
Owiredu WK et al [18]	B*	**B***	**A***	**A***	**A***	**-**	A*	**A***	**7**
Pasipanodya Ian etal [47]	A*	**A***	**-**	**A***	**A***	**-**	A*	**A***	**6**
Seid A et al. [42]	**A***	**A***	**A***	**B***	**A***	**A***	A*	**A***	**8**
Ugwumba FO etal [41]	A*	**B***	**B***	**A***	**-**	**B***	A*	**A***	**7**
Walle B	**A***	**A***	**A***		**B***	**B***	A*	**A***	**6**
Webb EM etal [39]	**A***	**A***	**A***	**A***	**B***	**B***	A*	**A***	**8**

Note: from each item account point. (Accept the study if total score ≥5)

Selection: (Maximum 5 stars)
1) Representativeness of the sample: a) Truly representative of the average in the target population. * (all subjects or random sampling) .b) Somewhat representative of the average in the target population. * (nonrandom sampling) .c) Selected group of users.d) No description of the sampling strategy.
2) Sample size:a) Justified and satisfactory. *.b) Not justified.
3) Non-respondents: a) Comparability between respondents and non-respondents characteristics is established, and the response rate is satisfactory. * .b) The response rate is unsatisfactory, or the comparability between respondents and non-respondents is unsatisfactory. c) No description of the response rate or the characteristics of the responders and the non-responders.
4) Ascertainment of the exposure (risk factor): a) validated measurement tool. ** .b) Non-validated measurement tool, but the tool is available or described.* c) No description of the measurement tool.
Comparability: (Maximum 2 stars)
1) The subjects in different outcome groups are comparable, based on the study design or analysis. Confounding factors are controlled. a) The study controls for the most important factor (select one). * b) The study control for any additional factor. *
Outcome: (Maximum 3 stars)
1) Assessment of the outcome: a) Independent blind assessment. **,b) Record linkage. **,c) Self report. *,d) No description.
2) Statistical test:a) The statistical test used to analyze the data is clearly described and appropriate, and the measurement of the association is presented, including confidence intervals and the probability level (p value). *,b) The statistical test is not appropriate, not described or incomplete
